# Spontaneous ventral urethral fistula in a young diabetic man: a case report

**DOI:** 10.1186/1752-1947-1-80

**Published:** 2007-09-05

**Authors:** Stefan Denzinger, Wolf F Wieland, Maximilian Burger, Wolfgang Otto

**Affiliations:** 1 Department of Urology, University of Regensburg, St. Josef's Hospital, Landshuterstr. 65, 93053 Regensburg, Germany

## Abstract

We present the first case reported in the medical literature of a patient with a spontaneous ventral urethral fistula accompanied by severe infection due to diabetes mellitus. A 34-year-old man with poor controlled adult-onset diabetes mellitus was admitted to our hospital with a large subcutaneous abscess involving the complete penis, scrotum and perineum. The patient did not report any history of any penile trauma or local infection but has experienced transient swelling of the perineal region following urination. Initial surgical treatment consisted of surgical debridement of necrotic tissue. At this time reconstructive surgery was impossible and a suprapubic cystostomy was performed. After 4 months of suprapubic urinary diversion the urethral fistula resolved and function of external genitalia was reestablished. In a follow-up period of 40 months no recurrence occurred. Spontaneous diabetes-associated ventral urethral fistulas are extremely rare and we are not aware of any other published case report.

## Background

Formation of an urethral fistula is a rare event and is usually a result of infectious complications or due to injury or surgery [1,2]. Acquired cases have been reported after blunt penile trauma [3] or straddle injury [4], but development of fistulas remains exceptional even under these circumstances. Even after complex hypospadia repair it is reported in no more than about 10% of cases [5]. Congenital urethral fistulas are seen as rare anomalies, usually in combination with anorectal malformations [6]. To our knowledge however no case of a spontaneous ventral urethral fistula has been reported.

We present the case of a patient with an urethral fistula in the absence of any of the common causes. In this case this complication seems to be related to poorly controlled diabetes. In patients with diabetes surgical therapy can be more challenging due to impaired wound healing.

## Case presentation

A 34-year-old male patient was admitted to our department in January 2004 complaining of a painless penoscrotal swelling immediately following urination. The swelling, which decreased after about one hour, had been observed over the past week. Two days before undergoing medical treatment the patient had experienced fever and some local pain, however no dysuria was noted.

On examination we found the entire scrotum and perineum swollen to a remarkable size of about 15 cm in diameter. In addition pus draining out from a perineal bump was noted. No penile, genital, truncal or facial anomalies were noted or had been known. The patient assured that there had been no trauma or foreign body insertion into the urethra. His medical history included poor controlled non insulin-dependent diabetes mellitus initially diagnosed about three months earlier. Otherwise the patient was in good medical condition. Serum glucose level was 20.2 mmol/l, haemoglobin A1C was 11.2%, white blood cell count was 18,600/μl. Urine analysis was normal.

**Figure 1:**
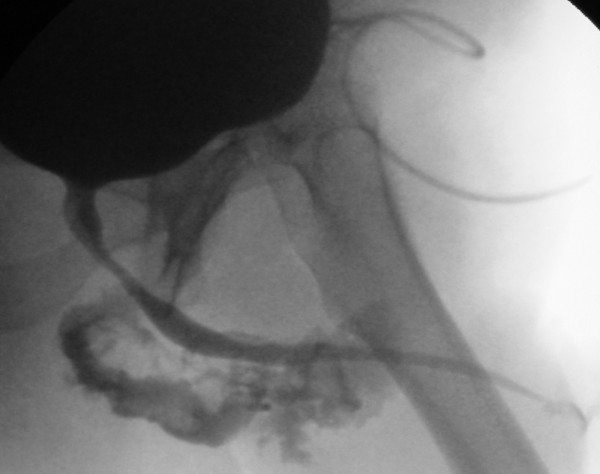
The preoperative retrograde urethrogram shows urethral leakage.

**Figure 2:**
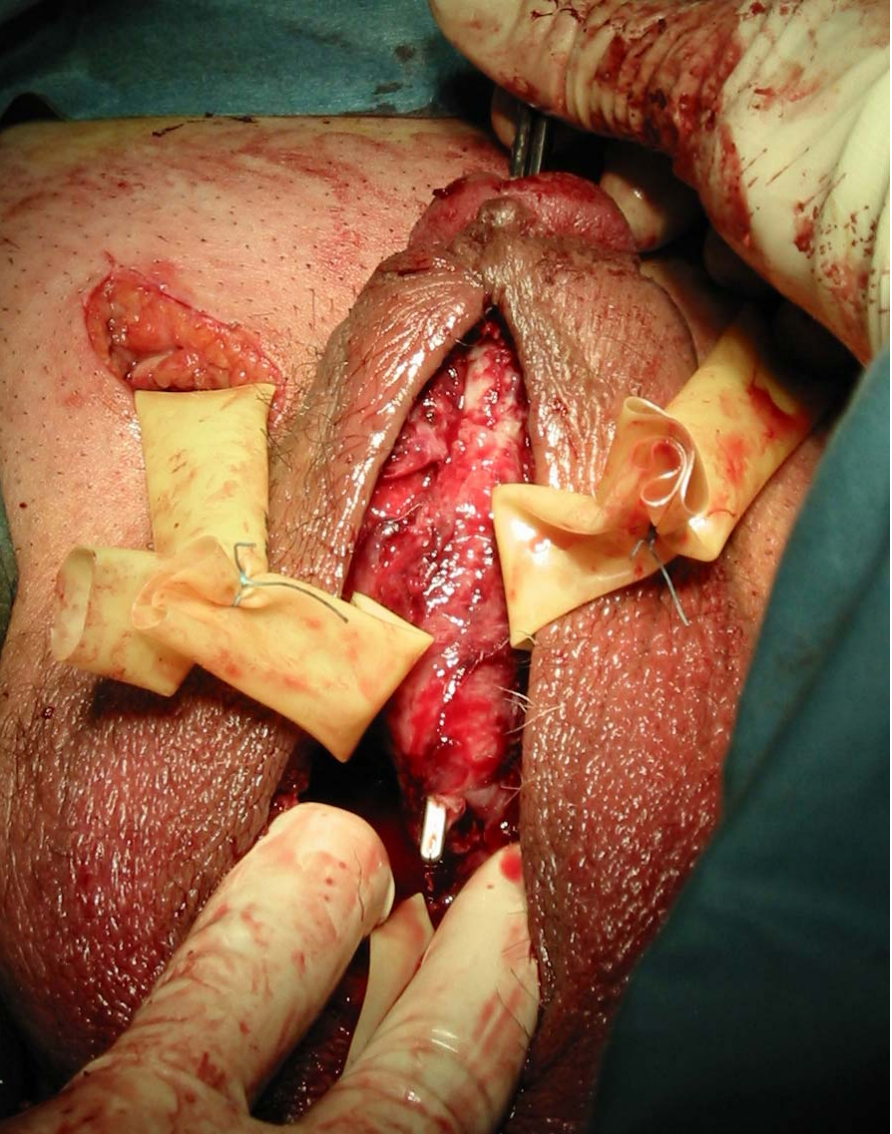
Intraoperative view: the fistula on the ventral side of the bulbar urethra is marked with a white stick.

As the patient exhibited signs of progressive local sepsis broad spectrum antibiotic treatment was initiated. Preoperative retrograde urethrogram confirmed a ventral bulbar urethral fistula (fig. 1). Initial surgical treatment under general anaesthesia included perineal, scrotal and penile debridement. Approximately 500 ml of pus were evacuated from the abscess cavity. Microbiologic analysis confirmed growth of mixed anaerobe bacteria. Intraoperative exploration identified a fistula, about 5 mm in diameter, on the ventral side of the bulbar urethra (fig. 2). As reconstructive surgery could not be undertaken, the patient underwent daily local debridement under local anaesthesia and suprapubic urinary diversion accompanied by strict control of his diabetes. After three weeks the man was discharged following complete secondary wound healing. Four months later no urethral leakage was visible upon repeat retrograde urethrogram. At this time the suprapubic catheter was removed and the patient was able to urinate without further problems. High-pressure voiding was excluded by urodynamic evaluation. The patient developed a painful scar on the ventral aspect of the penis in August 2004 and underwent resection of this tissue with good cosmetic and functional result. Since then the patient has presented regularly in our department and has shown no signs of recurrence in a follow-up period of 40 months.

## Discussion

Urethral fistulas are rare and their pathogenesis is usually due to infections, injuries or previous surgery e.g. urethroplasty [1,2]. Our patient probably developed a subclinical infection in a periurethral gland or a congenital asymptomatic urethral diverticulum, leading to periurethral abscess, which progressed to necrotizing fasciitis in the face of a poorly controlled diabetes mellitus [7]. One case with a less extensive dorsal urethral fistula, which was successfully treated by fistula incision and long term suprapubic urinary diversion, has been published [8]. In the case presented here complete excision and surgical reconstruction were virtually impossible without affecting urinary continence due to the position of the fistula. Closure of urethral fistulas is quite difficult owing to thin dermal layer. Furthermore there is a substantial risk of erectile dysfunction and scar formation. To our knowledge the case of a ventral urethral fistula has not previously been reported. Radical surgery with complete excision of the affected tissue may not be the treatment of choice in every urethral fistula. We have shown that in such an extensive case surgical debridement of necrotic tissue with postoperative suprapubic urinary diversion can be successful. Normalization of serum glucose level by oral medication or insulin therapy is the basis of every treatment of diabetes related complications.

## Conclusion

In this case report we present details of diagnosis and treatment as well as therapy results and details of follow-up of a 34-year-old diabetic man who had developed an urethral fistula. Spontaneous ventral fistulas of the urethra are extremely rare and to our knowledge have never been reported. Whereas urethral fistulas appear more often after accidents, such as blunt penile trauma or straddle injury, spontaneous forms may be due to infection in the presence of diseases of metabolism. In this context the poorly controlled diabetes of our patient was believed to be responsible for the development of this ventral urethral fistula. Reestablished function of the external genitalia without any signs of fistula recurrence after a follow-up period of more than three years show that such rare and severe pathologies of the urogenital tract can be treated successfully by a combination of medical and surgical means.

## Competing interests

The author(s) declare that they have no competing interests.

## Authors' contributions

WO drafted the manuscript. SD and MB treated the patient and helped to draft the manuscript. WFW supervised treatment and drafting of the manuscript. All authors read and approved the final manuscript.
